# Primary Signet-Ring Cell Adenocarcinoma of the Urinary Bladder Treated with Partial Cystectomy: A Case Report and Review of the Literature

**DOI:** 10.1155/2017/6829692

**Published:** 2017-12-11

**Authors:** Umesh Jayarajah, D. M. Hilary Fernando, Kasun Bandara Herath, M. V. Chandu de Silva, S. A. S. Goonewardena

**Affiliations:** ^1^Department of Urology, National Hospital of Sri Lanka, Colombo, Sri Lanka; ^2^Department of Pathology, Faculty of Medicine, University of Colombo, Colombo, Sri Lanka

## Abstract

Primary signet-ring cell carcinoma is a variant of adenocarcinoma which is extremely rare, associated with poor prognosis and generally found to be resistant to chemotherapy and radiotherapy. We report a case of primary signet-ring cell carcinoma of the bladder which was successfully treated with partial cystectomy. A 71-year-old female with a history of type 2 diabetes, hypertension, and ischaemic heart disease presented with painless haematuria for 2 months' duration. The abdominal ultrasonography showed a localised polypoidal vesical growth arising from the bladder dome. Cystoscopy revealed an exophytic solid tumour in the anterior fundal wall. A deep transurethral resection of bladder tumour was done and histology revealed an adenocarcinoma composed of mucinous and signet-ring cell components. Later, considering the patient's age and the poor general condition, a partial cystectomy was done. Follow-up cystoscopy and ultrasonography were done at 12 months and there was no evidence of tumour recurrence and the patient is currently symptom-free. Partial cystectomy may be considered in patients with localised tumour without evidence of metastasis and poor general condition. Regular cystoscopies and ultrasound imaging are necessary for follow-up and early identification of recurrences.

## 1. Introduction

Bladder cancer is the 9th most common cancer in the world [1]. Histologically, about 90–95% of bladder cancers are of urothelial origin [2]. The incidence of adenocarcinoma of the bladder represents only 2% of all epithelial bladder tumours. Primary signet-ring cell carcinoma is a variant of adenocarcinoma which is extremely rare with estimated prevalence of 0.24% of primary bladder cancers [3, 4]. It was first described by Saphir in 1955, and less than 200 cases have been reported in the literature since then [5, 6]. It is associated with a poor prognosis and is generally found to be resistant to chemotherapy and radiotherapy [7]. We report a case of primary signet-ring cell carcinoma of the bladder treated with a partial cystectomy.

## 2. Case Presentation

A 71-year-old female presented with a history of intermittent episodes of painless haematuria with passage of clots for 2 months' duration. She had a past history of type 2 diabetes mellitus, hypertension, and ischaemic heart disease. There were no other lower urinary tract symptoms and there was no family history of malignancies. The general and abdominal physical examinations were unremarkable. Haemoglobin was 8.6 g/dl on admission and required blood transfusions. Other basic biochemical parameters including serum creatinine were normal. The transabdominal ultrasonography showed a polypoidal vesical growth measuring 2.9 × 2.5 × 2.4 cm arising from the posterolateral wall of the bladder dome. The pre- and postvoid volumes were 180 ml and 44 ml, respectively. Both kidneys and upper urinary tracts appeared normal. Cystoscopy revealed an exophytic solid tumour measuring 1.5 × 2.0 cm arising from the anterior fundal wall covered with slough. A deep transurethral resection of bladder tumour was done.

The histopathological analysis revealed an adenocarcinoma composed of mucinous and signet-ring cell components. Thus, a secondary adenocarcinoma or a rare variant of primary adenocarcinoma of the bladder was suspected.

We performed a complete gastrointestinal endoscopic evaluation to exclude an extravesical primary tumour site, which was found to be normal. Later, considering the patient's age and the poor general condition, a partial cystectomy was done through a Pfannenstiel incision. The tumour was found to be adherent to part of the rectus abdominis muscle. Peritoneum was opened into and a generous margin of excision was achieved. No obvious pelvic lymphadenopathy was noted. The recovery following surgery was uneventful.

**Figure 1:**
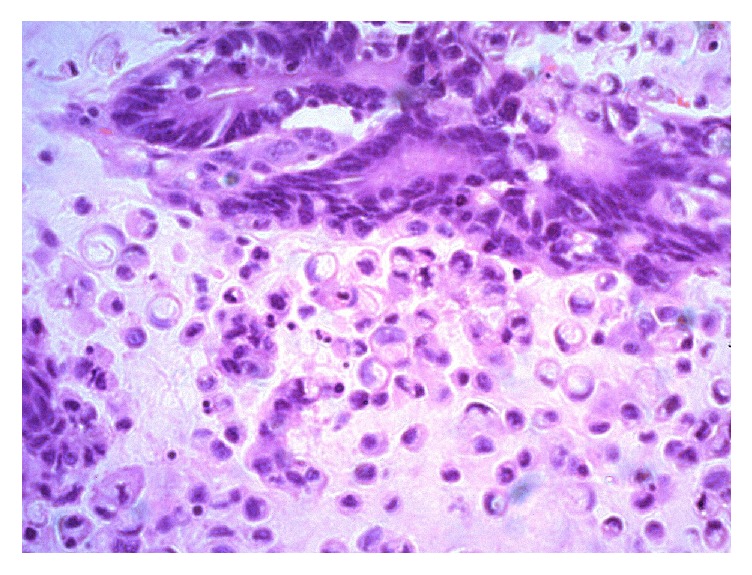
H&E staining viewed under ×40 showing a bladder carcinoma composed of mucinous and signet-ring cell components, showing nests of columnar cells and signet-ring cells floating in pools of extracellular mucin.

Macroscopic examination revealed an ulcerated mucosa. Cut surface revealed an intramural cystic mass with glistening surface measuring 21 × 15 × 15 mm. Histopathological evaluation revealed a primary bladder carcinoma composed of mucinous and signet-ring cell components, showing nests of columnar cells and signet-ring cells floating in pools of extracellular mucin (Figure 1). The columnar cells contained pleomorphic hyperchromatic nuclei. Signet-ring cells contained intracytoplasmic mucin. The epithelium was ulcerated and inflamed and was focally continuous with the tumour. The tumour invaded the adjacent muscular layer. There was no evidence of lymphovascular or perineural invasion. The tumour was 1.5 mm away from the deep and lateral margins.

**Figure 2:**
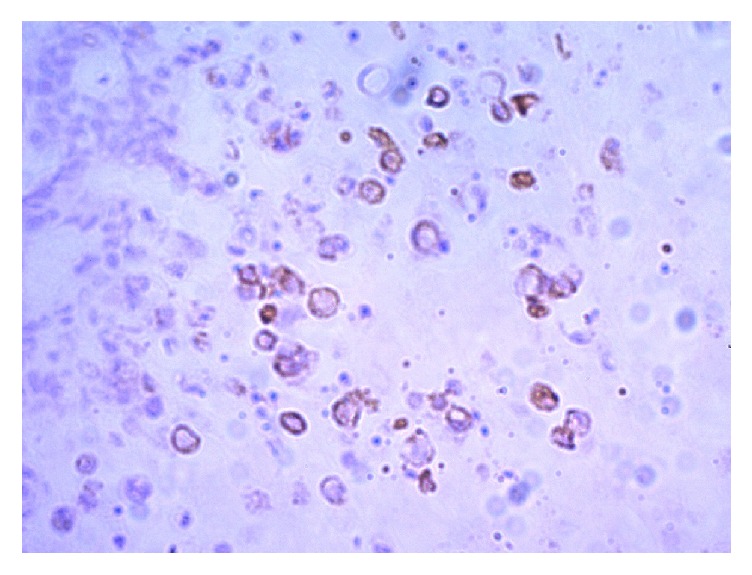
Immunohistochemical analysis with Cytokeratin 7 under ×40 showing focal cytoplasmic positivity.

Furthermore, immunohistochemical analyses showed focal cytoplasmic positivity for Cytokeratin 7 (Figure 2) and Cytokeratin 20 in tumour cells. Also, tumour cells showed nuclear positivity for CDX2 and cell membrane positivity for beta-catenin with no nuclear staining. Thus, in our patient, the immunohistochemical profile with the aid of negative endoscopy was consistent with a primary adenocarcinoma of the bladder rather than a metastatic deposit from colon. Follow-up cystoscopy and ultrasonography were done at 12 months and there was no evidence of tumour recurrence and the patient is currently symptom-free.

## 3. Discussion

Adenocarcinoma of the bladder accounts for only 0.5%–2% of all primary malignant tumours of the bladder [4, 8]. Most adenocarcinomas of the urinary bladder are secondary tumours resulting from direct extension from adjacent organs such as colon, prostate, and female genital tracts or metastatic spread via haematogenous and lymphatic route from distant organs such as stomach, lung, and breast [9, 10]. This variant is a poorly differentiated form of adenocarcinoma and its incidence represents about 0.24% of bladder cancers [9].

Haematuria is the most commonly described clinical presentation, which is also the presentation of this patient. However, other forms of presentation were also described such as dysuria, day-time urinary frequency and urinary incontinence or retention [11].

The histologic diagnosis is based on the presence of characteristic signet-ring cells filled with cytoplasmic mucin-containing vacuoles compressing and displacing the nucleus into a peripheral crescent alongside the cell wall. The component of signet-ring cells in the tumour is variable and it was reported as greater than 75% in almost half the cases [12].

Distinguishing this carcinoma from metastases is essential in the management as the treatment strategies required are entirely different. Furthermore, the histological appearance of primary signet-ring cell carcinoma of the urinary bladder is the same as that of other sites. Thus, evaluation of other primary sites is mandatory in view of excluding a metastasis [13]. In a female patient, possible primary tumours include tumours from colon, breast, and genital system that should be considered as differential diagnosis [10]. This is because primary signet-ring cell carcinoma has been reported to occur in the cervix and particularly the ovary which are closely related to the bladder [14, 15].

In our patient, the clinical imaging and immunohistochemistry were compatible with primary signet-ring cell cancer of the bladder. In the reported case, tumour tissue stained positive for CK7, CK20, and CDX2. Furthermore, cell membrane positivity for beta-catenin was seen with no nuclear staining. Although immunohistochemistry may be helpful in the diagnostic work-up of the cell types, it is difficult to distinguish between primary and secondary adenocarcinoma [16]. However, strong nuclear staining on beta-catenin was seen in the majority of metastatic colonic adenocarcinomas, while the positivity in primary bladder adenocarcinoma was reported to be low, which may be helpful in distinction between the two [16]. Due to these limitations, clinical and radiological correlation is mandatory in the distinction between primary and secondary adenocarcinoma of the bladder before rendering a histopathological diagnosis [16].

Signet-ring cell carcinoma is known to be associated with poor prognosis. At the time of diagnosis, distant metastases are seen in about 25% of patients and stage IV disease is seen in almost half the patients. This is due to the insidious progression of the disease [17]. The treatment of the disease is challenging, when it is diagnosed at an advanced stage. Surgery is the mainstay of treatment and usually radical cystectomy is done. Often, the reported cases have been shown as incomplete resection with no clear margins on the specimen [18]. Considering the rarity of this histologic type of tumour, there is no consensus regarding the management after surgical care. Chemotherapy and radiation therapy are discussed.

However, in this case report, partial cystectomy was done following a deep transurethral resection of bladder tumour (TURBT). Follow-up cystoscopy and ultrasonography at 12 months have shown no evidence of tumour recurrence. Partial cystectomy may be considered in selected patients with a localised tumour and poor general condition who are less likely to withstand a major surgery such as radical cystectomy. Regular cystoscopies and ultrasound imaging are necessary for follow-up and early identification of recurrences. Chemotherapy in such patients may be reserved for those with recurrence detected at cystoscopy.

## 4. Conclusion

The primary signet-ring cell carcinoma of the bladder is a rare tumour known to be associated with poor prognosis. However, in patients with localised tumours without evidence of metastasis and poor general condition, partial cystectomy may be considered. Regular cystoscopies and ultrasound imaging are necessary for follow-up and early identification of recurrences.
